# Mechanical and Failure Behavior of Soft-Hard Composite Rock with Three Parallel Joints Under Uniaxial Loading: Insights Based on AE and DIC Techniques

**DOI:** 10.3390/ma18051088

**Published:** 2025-02-28

**Authors:** Chaoyi Yang, Su Li, Xinglong Feng, Lianrong Wu, Hang Lin

**Affiliations:** 1 School of Resources and Safety Engineering, Central South University, Changsha 410083, China; yangchaoyiyn@126.com (C.Y.); lisu1996@csu.edu.cn (S.L.); 2 Yunnan Diqing Non-Ferrous Metals Co., Ltd., Shangri-La 674400, China; fengxinglongyn@126.com (X.F.); wuliangrongyn@126.com (L.W.)

**Keywords:** jointed soft-hard composite rock, uniaxial compression, DIC, AE, crack initiation mechanism

## Abstract

Jointed soft-hard composite rocks are frequently encountered in nature, and this complex structure contributes to unpredictable fracturing mechanisms and failure behavior. In this study, soft-hard composite rocks with three joints were fabricated to conduct a uniaxial loading experiment, supplemented by Digital Image Correlation (DIC) and Acoustic Emission (AE) experiments. The results indicate that the mechanical parameters display a V-shape variation trend with the increase of joint angle, which minimized at 30°. The peak strength ranges from 33.48 MPa to 44.93 MPa. The failure characteristics change from tensile failure to shear failure and finally to intact failure. According to the displacement curves on both sides of the crack, the initiation of wing cracks is driven by the direct tensile displacement field and indirect tensile displacement field for specimens with joint angles of 0–30° and 75–90°, respectively. While the crack initiation from joint tips corresponding to specimens with a joint angle of 45–60° is controlled by direct and indirect tensile displacement fields. Wherein the cracks initiate from the coplanar joint in the hard layer, driven by the indirect tensile displacement field, and the cracks expanding upward from other joint tips are more susceptible to the indirect tensile displacement field.

## 1. Introduction

Soft-hard composite rocks are commonly observed in engineering. The difference in mechanical properties between the different rock layers will lead to stronger anisotropy compared to homogenous rock [1,2,3]. Under external loads, new cracks tend to develop in the joints, pores, and other initial defects of soft-hard composite rocks. The expansion and coalescence of cracks will further reduce the strength and stability of the composite rocks, posing a great threat to engineering and personal safety. Therefore, the study of mechanical performances of soft-hard composite rocks with joints is of great significance.

The failure of engineering rock mass is often originated from the expansion and coalescence of cracks between joints, and therefore understanding the relevant mechanism facilitates the avoidance of engineering disasters and the selection of reinforcement measures [4,5,6,7,8,9]. Numerous scholars have performed systematic and in-depth research on this issue. Subjected to external loads, tensile cracks tend to emerge first at the joint tip, called wing cracks [10,11]. In addition, the cracks are named according to the initiation location and the expansion direction of the crack [12]. In order to study the coalescence mechanism between joints, scholars have conducted experiments on double-jointed rock mass and investigated the effects of changes in joint geometry (joint angle, ligament angle, and ligament length, etc.) on the crack behavior, which turned out that the increase in rock-bridge distance is detrimental to the coalescence behavior, and the increase in rock-bridge inclination induces a gradual change in the coalescence mode between cracks from shear to tensile [13,14,15,16]. Moreover, the strength of the rock mass also plays an important role in the coalescence pattern [17,18]. Under the influence of long-term geological tectonics, joints in natural rock mass tend to occur in pairs, with similar scales and orientations. In this case, physical experiments have been carried out on three, four, and multi-jointed rock masses in an attempt to clarify the failure behavior of rock masses with more complex joint distributions [19,20,21,22,23]. It was found that the coalescence between joints is closely related to the joint angle and ligament angle, and is more likely to occur between two joints with larger rock bridge inclination. The observed crack coalescence patterns between multi-jointed rocks are similar to those between three joints [24]. During this process, the DIC technique was widely used since the cracking behaviors are hardly observed by the naked eye, which can well reproduce the crack extension by comparing the change of scattered spots on the rock surface. Via this method, Li et al. [25] analyzed the change of crack types with joint geometry in detail and found that with the increase of joint angle, the out-of-plane portion of horsetail-like cracks at the outer end of the joints will gradually change from tensile cracks to shear cracks. Wang et al. [26] analyzed the scattering pictures by Ncorr comparison to determine the direction of the movement of cracks on both sides of the joint tips. All the above studies have enriched our understanding of crack initiation, extension, and coalescence in jointed rocks.

On the other hand, although there have been a large number of in-depth studies on the mechanical behavior of soft-hard composite rocks under tension, shear, static compression, and dynamic compression, the existence of joints greatly alters the failure behavior of soft-hard composite rocks and warrants further study [27,28,29,30,31]. Ma et al. [32] investigated the mechanical properties and failure behavior of soft-hard composite rocks with single joints by means of numerical simulation and analyzed the effects of joint location, joint angle, and joint size. Lin et al. [33] carried out uniaxial compression experiments on soft-hard composite rocks with parallel joints and evaluated the effects of joint angle (30–90°) and ligament angle (60–120°) on crack coalescence behavior, summarizing 7 coalescence modes. For the soft-hard composite rocks with more complex joint distribution, Lin et al. [34] studied the coalescence behavior in soft-hard composite rocks with three joints (the joint angle varies from 30° to 60°) and discovered that the addition of the third joint has a greater effect on the failure of the soft-hard composite rocks. The coalescence mode is more complicated than that of two-jointed soft-hard composite rocks, and ten coalescence characteristics were proposed. Zhao et al. [35] studied the mechanical behavior of multi-jointed soft-hard composite rocks under uniaxial compression and analyzed the influence of the distribution of the joint number on the failure of soft-hard composite rocks.

Based on the above analysis, just a few studies have been conducted on jointed soft-hard composite rock containing more than two joints and analyzed the influence of joint geometric parameters in detail. In this paper, soft-hard composite rock with three parallel changes from horizontal to vertical was employed to perform uniaxial compression experiments, and DIC and AE techniques were applied to assist in the analysis of the macro and micro failure characteristics. The influence of joint angle variation on the failure behavior and crack initiation mechanism of jointed hard-soft composite rocks was discussed. The research findings can provide references for disaster prevention in related rock mass engineering.

## 2. Experimental Schemes

### 2.1. Specimen Fabrication

**Figure 1:**
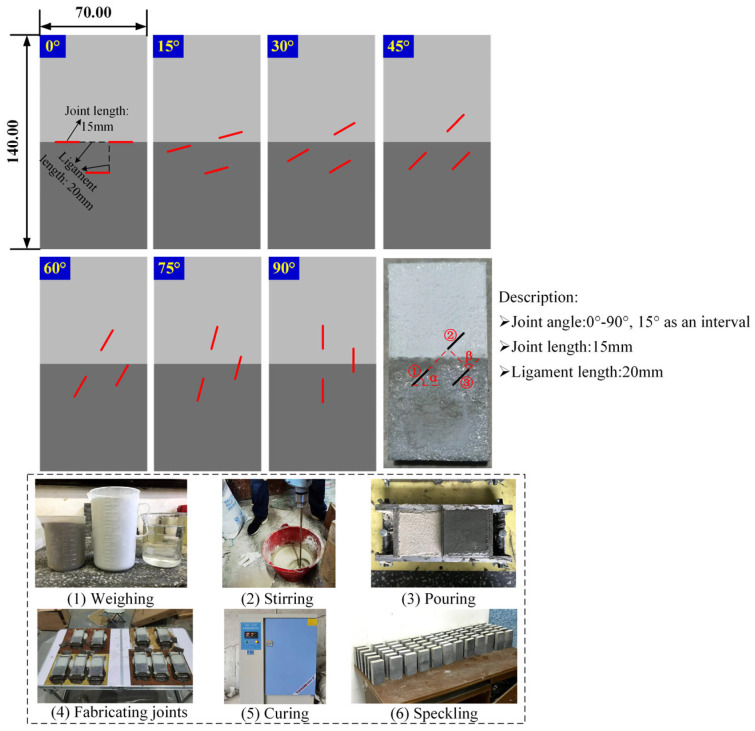
Jointed soft–hard composite rock specimens and fabrication process.

The sizes of the jointed soft-hard composite specimens prepared in the paper were 140 mm × 70 mm × 30 mm, as shown in Figure 1, with an aspect ratio of 2:1 according to the recommendation of ISRM [36]. To achieve a good layering effect, a slot was made in the middle of the long side of the mold, which was used to separate two cement mortars with different mechanical properties by inserting an iron sheet. The cement mortar used to simulate soft rock consists of PW325 white cement, fine sand, and water, and the cement mortar corresponding to hard rock is composed of PC425 black cement, fine sand, and water, both of which were mixed according to a mass ratio of 5:3:2. After pouring the cement mortars into the molds, putting an acrylic sheet with joint location information on the molds, and then prefabricated joints were prepared by inserting mica sheets with a height, width, and thickness of 30 mm, ×15 mm, and ×0.4 mm. 24 h later, the molds were demolded, and the specimens were placed in a curing box for a period of 28 days at 21.5 °C and 95% humidity. Due to the fact that DIC and AE techniques were also employed in these experiments, it was necessary to spray spots on the specimen surfaces and affix iron sheets to the sides of the specimens to facilitate the mounting of the AE probe adsorption fixtures. In this experiment, the variable is the joint angle, i.e., the angle between the joint and the horizontal direction (acute angle). The relative positions between the three joints were kept constant, and the fixed joint geometry parameters were presented in Figure 1. For ease of analysis, the coplanar joints were named as Joints ①②, and the parallel joints were named as Joints ③. The specimen was remarked by UT-*a*, wherein UT stands for uniaxial test, and *a* is joint angle. To avoid experimental error, three parallel specimens with the same joint geometric parameters were fabricated. Moreover, standard specimens of both types of cement were also prepared to obtain their basic mechanical parameters according to the recommendation of ISRM [37].

### 2.2. Equipment and Loading Scheme

**Figure 2:**
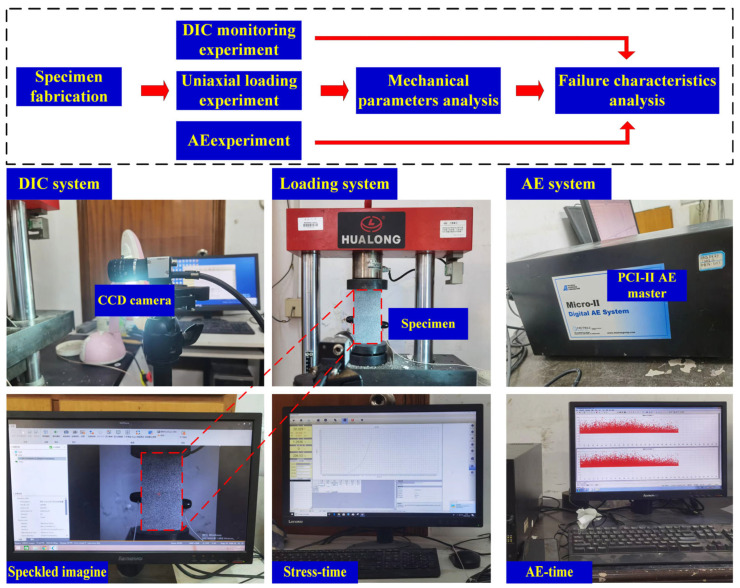
Flowchart and layout of the experimental apparatus.

As can be seen from Figure 2, the experimental equipment consists of two parts. One is the loading system, which is composed of Hualong compression machinery (HUALONG, Shanghai, China) and a control computer. The maximum loading force of the instrument is 300 kN, which meets the requirements of the experiment. The control system can provide a variety of loading modes, and the displacement-controlled loading mode was adopted in this paper with a loading rate of 0.003 mm/s. Additionally, a DIC system and an AE system (PAC, Princeton Junction, NJ, USA) were also utilized to monitor the surface strain field changes and acoustic emission events of the specimens during the loading process, respectively. The DIC system (WORKPOWER, Shenzhen, China) includes a CCD camera, a light source, and a control unit. Before the experiment, the aperture and focal length of the camera were continuously adjusted to record the scattering spots as clearly as possible. In order to completely record the crack propagation process, the shooting speed was set to 15 pictures/s. To ensure the accuracy of DIC results, during the analysis process, the subset is set to 21, and the step is set to 3 [25]. The latter is made up of an acoustic emission host, an acoustic emission transducer, and a front-end amplifier. The threshold of the AE signal is 40 dB. When the stress is applied to the end of the specimen, the acoustic emission signals and scattering figures are recorded simultaneously until rock failure.

## 3. Analysis of Experimental Results

### 3.1. Mechanical Curves

**Figure 3:**
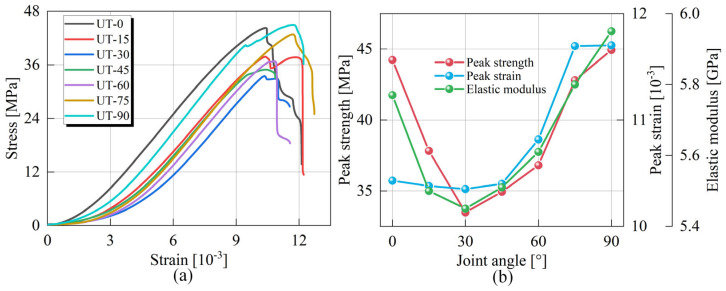
Mechanical response of jointed soft-hard composite rock under uniaxial loading: (**a**) Stress-strain curves, (**b**) Mechanical parameter curves.

Figure 3 presents the complete stress-strain curves and mechanical parameter variation curves of the soft-hard composite rock specimens containing three joints under uniaxial loading. In Figure 3a, each stress-strain curve clearly shows four different stages, which are the compression stage, elasticity stage, crack extension stage, and post-peak stage. The compaction stage mainly corresponds to the compaction of primary voids, and the curves display an up-concave shape. The specimen will undergo a large deformation under the action of a very small load. For the crack extension stage, the tensile cracks on the tip of joints will be initiated and expanded, and therefore it is easy to see that there is a slight stress drop in some curves, which is mainly due to the stress release caused by the sudden and rapid expansion of the tensile crack. However, the integrity of the specimen remains sufficiently high so that the stress-strain curve continues to rise after a slight drop. The specimen experienced intense damage during the post-peak stage, mainly in the form of shear crack initiation and crack coalescence in the rock-bridge region, which significantly affected the integrity and stability of the specimens, causing the stress-strain curves to fall continuously. It should be noted that the post-peak stage morphology of the specimens with different joint angles varies, which is associated with the variation of the geometric parameters of the joints affecting the damage and coalescence characteristics of specimens. Figure 3b shows the mechanical parameter curves, including the variation trends of peak strength, peak strain, and elastic modulus. In general, the mechanical parameters of each specimen show a decreasing and then increasing trend with the increase of joint angle. The minimum value is obtained when the joint angle is 30°, and the maximum value is achieved when the joint angle is 90°. Specifically, in the interval [0°, 30°], the peak strength decreases from 44.23 MPa to 33.48 MPa, the corresponding strain decreases from 10.43 × 10^−3^ to 10.35 × 10^−3^, and the elasticity modulus decreases from 5.77 GPa to 5.45 GPa, with reductions of 24.30%, 0.77%, and 5.54%, respectively. Relatively, in the interval [30°, 90°], the peak strength increases from 33.48 MPa to 44.93 MPa, the corresponding strain increases from 10.35 × 10^−3^ to 11.70 × 10^−3^, and the elasticity modulus increases from 5.45 GPa to 5.95 GPa, with an increase of 34.24%, 13.04% and 9.17%, respectively.

### 3.2. Crack Evolution Analysis

The DIC technique, as a non-contact optical monitoring tool, is known for its strong anti-interference, and its monitoring principle is to track the deformation process of the scattering pattern on the surface of the object and calculate the change of the gray value of the scattering domain so as to get the deformation and strain data of the measured object surface [38]. Through the generation of strain bands in the strain field, the extension can be a great way to understand the crack initiation, extension, and coalescence behavior of cracks in jointed rocks. Moreover, the arrangement of virtual extensometers on both sides of the cracks can accurately compare the normal and tangential displacements on the two sides of the cracks for a better qualitative analysis of the crack types. By using this technique, numerous scholars have successfully investigated the damage behavior of rocks under various loading conditions. On the other hand, the micro-failure behavior of the rock produces a subtle acoustic sound, which can be completely and accurately recorded at each loading stage with the help of a high-precision, high-frequency acoustic emission host. By analyzing the changes of acoustic emission parameters (ring count, cumulative ring count, b-value, tensile-shear signal [39]), it is possible to gain a deeper understanding of the failure mode of the rock.

**Figure 4:**
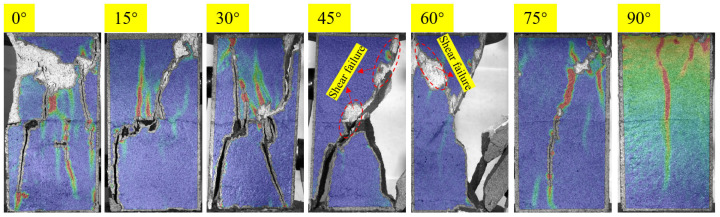
Failure morphology of specimens with different joint angle.

Figure 4 illustrates the final failure modes of joint soft-hard composite specimens with different joint angles. From the failure pattern and crack propagation paths, it can be noticed that the variation of a joint angle has a great influence on the failure modes of the specimens. Based on the crack type, the failure of specimens with joint angle of 0–30° is mainly caused by the tensile wing cracks initiated from the joint tips and tensile cracks generated in rock sections. As can be seen from the figure, for the specimens with a joint angle of 45–60°, the shear failure characteristics can be clearly observed in addition to the wing cracks initiated from the joint tips. For the specimens with a joint angle of 75–90°, the failure plane is roughly parallel to the loading direction, which is the same as the failure mode of intact rock. Therefore, specimens with a joint angle of 30°, 45°, and 75° were taken as examples to analyze the failure process of specimens by analyzing the evolution of the strain field and AE parameters during loading.

#### 3.2.1. Tensile Failure Mode: Specimens UT-30

**Figure 5:**
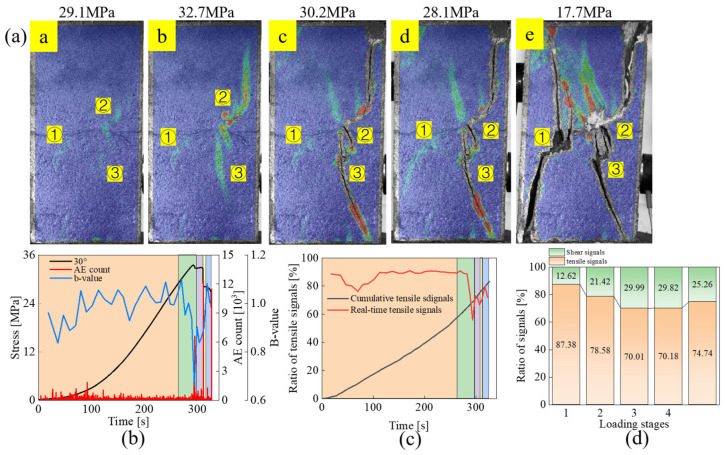
Evolution of strain field and acoustic emission parameters for specimen UT-30: (**a**) Strain field evolution, (**b**) AE count and b-value, (**c**) Proportion of tensile signals, (**d**) Tensile-shear signal proportions in different loading stages.

The strain field evolution, the acoustic emission parameters (AE counts, b-values) versus stress, and the variation of the tensile signal throughout the loading process are shown in Figure 5. When the stress reaches 29.1 MPa, the strain bands initiate, at the joint tips first, among which the expansion of the strain bands at the tips of the upper joints is more obvious. When stress increases to 32.7 MPa, vertical tensile strain bands appear outside the right tip of the upper joint ② and the left tip of the lower joint ③. According to the large decrease in the b-value present at this stage, an increase in the proportion of large damage events can be detected. This corresponds to the coalescence of the outer tensile strain band of joint ② with the outer tip of joint ②. Since the crack coalescence consists of an out-of-plane tensile crack and a coplanar shear crack, and the tensile strain band at the left tip of joint ③ extends towards the lower right corner of the specimen under shearing, there exists a certain degree of reduction in the tensile signal at stage *b* with respect to stage *a*. In stage *c*, the strain band at the left tip of joint ③ extends to the tip of joint ②, and coalescing occurs, resulting in local failure. The percentage of tensile signal further decreases to 70.01% in this stage. Compared with stage *c*, the change in stage *d* is mainly the generation of a strain band at the left tip of joint ①. Therefore, the difference in the percentage of tensile cracks is very small. With further loading, under the effect of shear slip, the strain band at the outer side of joint ① laps with the tip of joint ①, and the wing crack at the right tip of joint ① rapidly extends to the end of the specimen, causing final failure. The proportion of tensile signals in this stage rises to 74.74%, which means that the composition of the failure plane on the left side of the specimen is dominated by tensile cracks. Throughout the loading process, high AE count events occur in stages *b*, *c*, and *e*, corresponding to the coalescence of the tip of joint ② in stage *b*, the local failure caused by the coalescence of the tip of joint ② and ③, and the final failure of the specimen, respectively.

#### 3.2.2. Shear Failure Mode: Specimens UT-45

**Figure 6:**
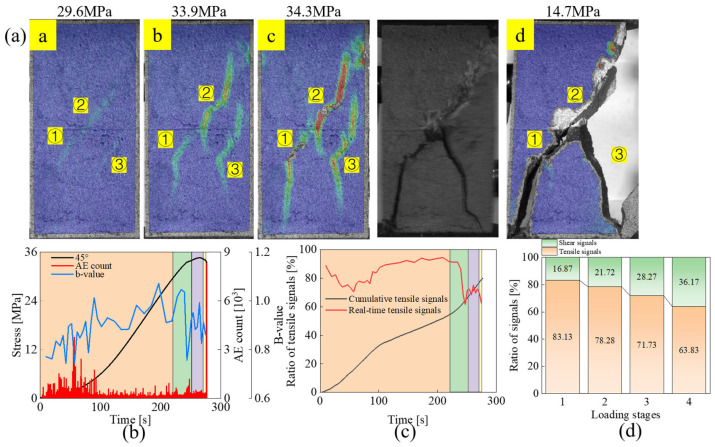
Evolution of strain field and acoustic emission parameters for specimen UT-45: (**a**) Strain field evolution, (**b**) AE count and b-value, (**c**) Proportion of tensile signals, (**d**) Tensile-shear signal proportions in different loading stages.

From Figure 6, it can be observed that the strain field first changes at the joint tips, especially the upper joint tips. However, the tensile strain bands at the joint tips corresponding to the wing cracks do not further expand with loading, but the out-of-plane tensile strain bands appear on the outside of the tips of both sides of the upper joint. When the stress nearly reaches the peak, the strain bands outside the tips of the upper joint turn red, representing the formation of macroscopic cracks. There are also vertical tensile strain bands initiated at the right interlayer face of the specimen. Compared with stage *A*, the percentage of tensile signals decreases from 83.13% to 78.28%, which represents that although the macro performance of the specimen in this stage is tensile strain band extension, the extension of macro tensile cracks is also affected by shear effects. After entering stage *c*, the specimen shifts from the pre-peak plastic deformation stage to the post-peak stage. The strain bands initiated from the tips of each joint extend further, and the out-of-plane tensile strain band near joint ② laps with the tip of joint ②. Furthermore, shear strain bands were also generated in the upper right corner of the specimen and in the rock bridge region between joint ① and ②, which also causes the increase of shear signal ratio in this stage. With loading, the shear strain band in the upper right corner of the specimen and the shear strain band in the rock bridge between joints ① and ② overlap with the joints, and shear damage occurs, forming a macro failure surface from the upper right corner to the lower left corner. Under the effect of shear slip, the strain band at the left tip of joint ② is tilted and extended to the tip of joint ③, and the vertical strain band at the interlayer surface of the specimen is extended to the right tip of joint ③, and further damage occurs in the lower part of the specimen. The ratio of tensile signal decreases to 63.83% at this stage, implying that shear microcrack is more important in the final damage stage. Compared with the specimens with 30° joint inclination, the specimens with 45° joint inclination exhibit more obvious shear damage characteristics. In terms of macro failure, there is surface spalling due to shear damage in the rock bridge area between joint ① and ② and on the right outer side of joint ②. Regarding the percentage of tensile-shear signals in each stage, although the percentage of tensile signals in the first few stages varies similarly, it increases from 70.18% to 74.74% in the last stage for the specimens with a joint angle of 30° while decreasing from 71.73% to 63.83% for the specimens with a joint angle of 45°, which proves that shear microcracks play a more significant role with the increase of joint angle.

#### 3.2.3. Failure Mode Similar to Intact Rock: Specimens UT-75

**Figure 7:**
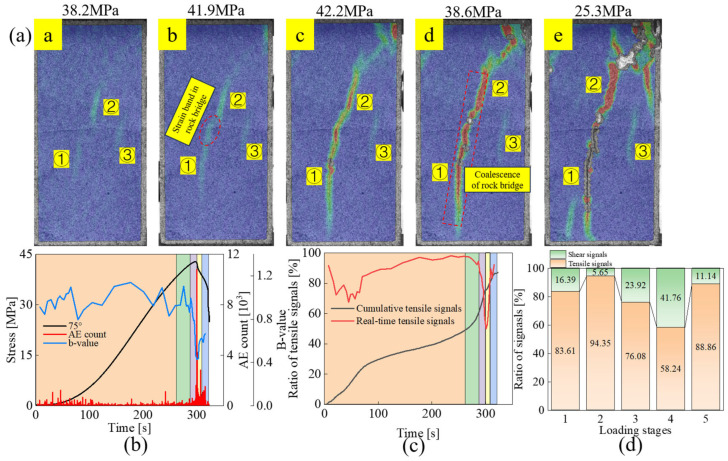
Evolution of strain field and acoustic emission parameters for specimen UT-75: (**a**) Strain field evolution, (**b**) AE count and b-value, (**c**) Proportion of tensile signals, (**d**) Tensile-shear signal proportions in different loading stages.

From the final morphology in Figure 7, the failure is composed of cracks initiated at the tips of joints ① and ②, and the change of the strain field at the tip of joints ③ is very weak. The strain field first initiates at the tip of each joint and further expands at joint ① and ② with the increase of stress. When the stress reaches 41.9 MPa, about 98% of peak strength, a strain band appears in the rock bridge region between joints ① and ②, as shown in stage *b*. According to Figure 7c, the percentage of tensile signals increases from 83.61% to 94.35%, indicating that tensile microcracks dominate in this stage. However, from the real-time percentage curve of tensile cracks, the tensile signal has been showing a decreasing trend between stage *b* and stage *d*, representing that the role of shear microcracks from stage *b* is increasing. In stage *c*, the stress-strain curve has entered the post-peak stage. The strain bands at the tips of joints ① and ② and the rock bridge region keep changing to red, meaning that macro cracks have been formed. Under the action of higher stress, the crack extension is not only composed of tensile crack alone but also shows a shear slip tendency along the joint. In addition, an inclined high-strain region develops in the upper right corner of the specimen. The proportion of tensile signal decreases to 76.08% at this stage and reaches a minimum of 58.24% at stage *d*, indicating that shear microcracks play a great role in crack propagation and failure plane formation throughout the post-peak stage. According to the evolution of the strain field, the coalescence between coplanar double joints occurs in stage *d*, where the tension cracks are always in an upward trend in this stage from the real-time tensile signal curve, confirming that the macroscopic failure of the specimen is mainly led by the tensile cracks.

## 4. Discussion

### 4.1. Macro Failure Characteristics

The shift in joint angle affected the specimen’s failure in an extremely important way. From Figure 4, the damage degree of the upper part of specimens with joint angle of 0–15° is greater than that of the lower part, but the failure plane is through the upper and lower layers of the specimen. When the joint angle is greater than 30°, the failure of the composite rock is closer to a hole. With the increase of joint angle (30–90°), the failure plane morphology of the specimens will also appear more concise. In the interval of 30–60°, its macroscopic shear damage characteristics will be more obvious. The failure plane can be divided into left and right parts, and the right failure surface is formed by the extended coalescence of the cracks initiated from the tips of joints. It is not difficult to find out that the coalescence pattern between joints is composed of tensile wing cracks. Apart from the coplanar shear cracks at the joint tips, the main components of the failure plane of the specimens are tensile wing cracks and out-of-face tensile cracks, which show obvious tensile damage characteristics. For specimens with joint angles of 45° and 60°, the propagation of cracks initiated from the tips of joints ① and ③ is more obvious, and the cracks coalesce in the rock bridge area, causing local failure on the right side of the specimen and leading to the final shear failure. There is obvious surface spalling accompanying the shear failure on the surface of specimens. When the joint angle reaches 75–90°, the fragmentation degree of the specimen is smaller, and there is only a failure plane coalescence along the coplanar joints, and the out-of-plane parallel joints are almost ineffective. For specimens with a joint angle of 75°, the cracks that constitute the failure surface are the tensile wing crack at the left tip of joint ② and the out-of-plane crack in the rock bridge area between the coplanar joints. For specimens with a joint angle of 90°, the failure plane is composed of cracks initiated from the coplanar joints.

**Figure 8:**
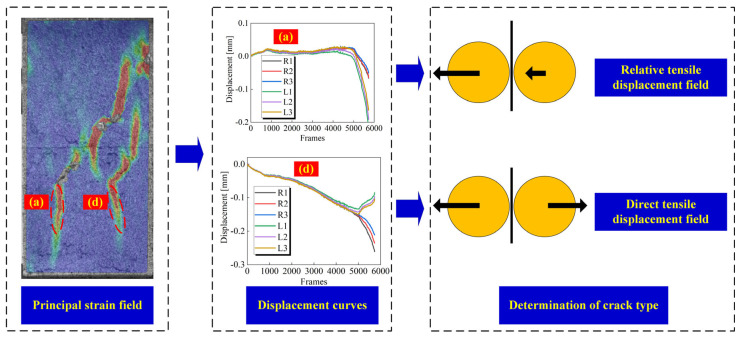
Method to determine the crack type based on the displacement curve of monitoring points.

In addition to the analysis of macro failure characteristics, the qualitative analysis of crack types can also help to understand the failure mechanism of jointed rock. In the strain field, monitoring points are arranged on both sides of the strain band, and the normal displacement of each monitoring point is extracted during the loading process. By comparing the trend of the displacement curves of the two monitoring points, it can be accurately assessed whether the crack extension is driven by the direct displacement field or the relative displacement field. When the displacement curves of the two sides of the monitoring point overlap well, it is considered that cracks have not yet been developed. Otherwise, crack formation is recognized. Moreover, it is possible to determine whether a macro crack is generated by checking the process of strain field changes. When the curves of monitoring points on both sides respectively move toward the positive and negative directions of the y-axis after generating relative displacement, it is identified that the crack is driven by the direct displacement field. While if they change in the same direction, the crack initiation and expansion of the crack are driven by the indirect tensile displacement field (Figure 8).

**Figure 9:**
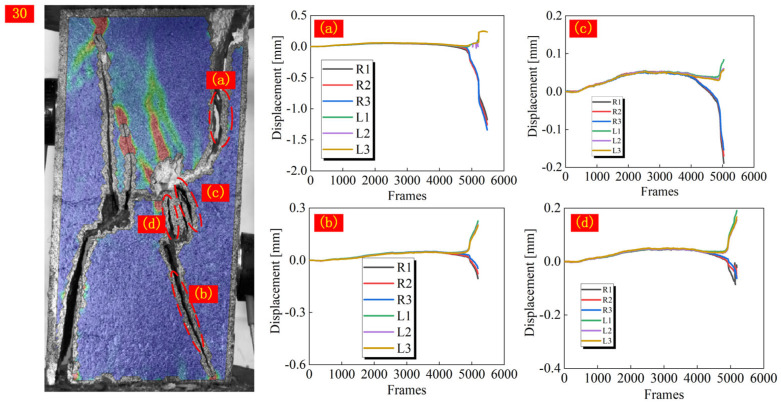
Trend of normal displacement on both sides of the crack in specimens with a joint angle of 30°.

**Figure 10:**
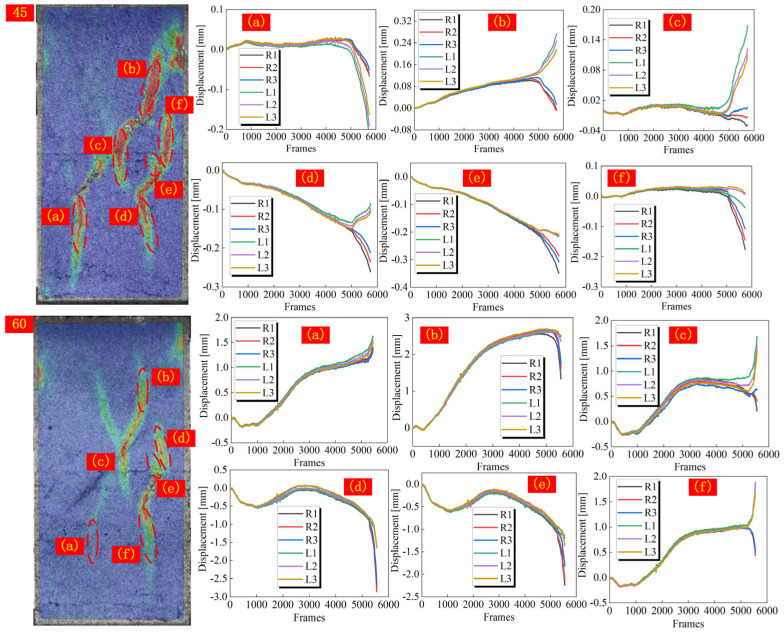
Trend of normal displacement on both sides of the crack in specimens with a joint angle of 45–60°.

**Figure 11:**
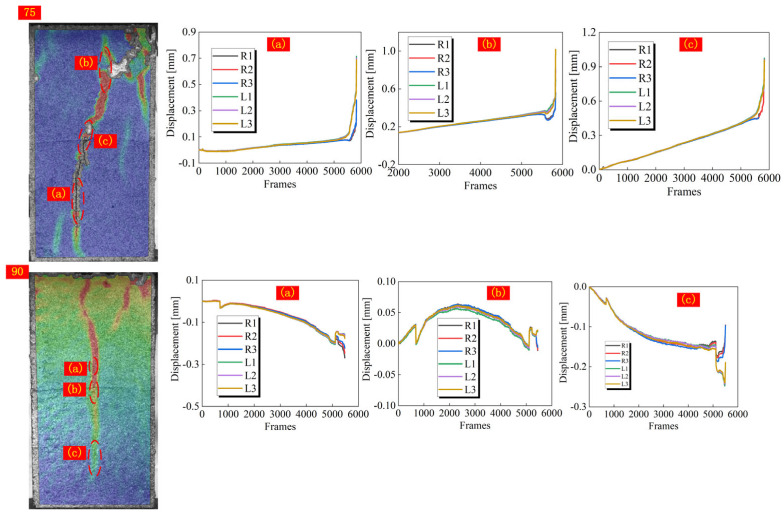
Trend of normal displacement on both sides of the crack in specimens with a joint angle of 75–90°.

According to Li et al. [25], when the joint angle of jointed homogeneous rock reaches 60°, the displacement field driving the initiation of cracks at the joint tip will change from direct tensile displacement field to indirect tensile displacement field. In this paper, the driving displacement field of cracks in specimens with a joint angle of 0–30° is consistent, so the specimens with a joint angle from 30° to 90° are selected for analysis. As shown in Figure 9, Figure 10 and Figure 11, the displacements on both sides of all measured cracks vary in both positive and negative directions when the joint angle is 30°, implying that the formation of cracks is all driven by a direct tensile displacement field. This is consistent with the conclusions obtained in rocks with a single lithology. When the joint angle is 45°, there is a difference in the driving field of the wing cracks at different joint tips. For joint ②, the right joint tip is driven by a direct tensile displacement field, and the left joint tip wing crack is initially driven by a direct tensile displacement field, which changes to an indirect tensile displacement field when the crack extends to the hard layer. For joint ③, the wing crack at the left joint tip is driven by a direct tensile displacement field, and the wing crack at the right joint tip is driven by an indirect tensile displacement field. Based on the macro crack expansion characteristics, the wing cracks at the left tip of joint ③ expand more rapidly than that of the right tip. This proves that the initiation and expansion of the wing cracks at the right tip are mainly driven by the indirect tensile strain field due to joint slip. When the joint slip does not generate significant changes, the crack expansion is slow. Beyond that, the wing cracks at both tips of joint ① are also driven by indirect tensile displacement sites. When the joint angle is 60°, the wing cracks at the tips of joint ③ are driven by direct and indirect tensile displacement fields, respectively, and the wing cracks at the tip of joint ① are caused by an indirect tensile displacement field, which is roughly the same as that of the specimen with a 45° joint angle. The difference is that the crack initiation and expansion of the crack in the right tip wing of joint ② is driven by an indirect tensile displacement field. When the joint angle rises to 75°, all cracks are driven by the indirect tensile displacement field, as can be seen from the relative displacements of the monitoring points on both sides of the cracks. For a 90° joint angle, the wing cracks at the joint tip are initially controlled by an indirect tensile displacement field, but the subsequent extension is controlled by a direct tensile displacement field. The strain band in the rock bridge region lags behind the joint tip, and thus its corresponding driving displacement field is the direct tensile displacement field. In summary, the displacement field driving the initiation of wing cracks from the joint tips shifts from a direct displacement field to an indirect displacement field as the joint angle increases. It is worth noting that the cracks in the rock bridge region of the specimen with a joint angle of 75° are driven by an indirect tensile displacement field, while the cracks in the rock bridge region of the specimen with a joint angle of 90° are driven by a direct tensile displacement field. This is mainly due to the fact that the strain band in the rock bridge region and the crack at the joint tip are almost initiated and propagated simultaneously for specimens with a joint angle of 75°, while the strain band in the rock bridge region lags behind the strain band initiated at the joint tip for specimens with a joint angle of 90°.

### 4.2. Micro-Failure Characteristics

**Figure 12:**
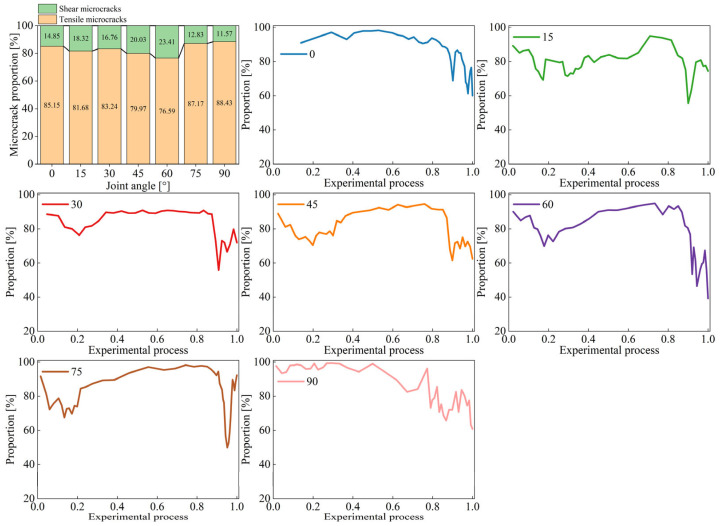
Tensile-shear microcrack percentage and real-time variation curve of tensile microcracks in specimens with different joint angles.

As illustrated in Figure 12, when the joint angle is in the range of 0–30°, the percentage of tensile microcracks shows a small fluctuation, which is weakly correlated with the joint angle, and the cumulative percentage of tensile cracks is above 80%. When the joint angle is 45–60°, the percentage of shear microcracks is increasing, which is consistent with the macroscopic crack analysis of the specimens with a joint angle of 45–60° showing more obvious shear failure characteristics on the failure plane. For specimens with a joint angle of 75–90°, the macrocracks mainly consist of wing cracks at the joint tips and cracks in the rock bridge region. When the rock bridge inclination is 75–90°, it is difficult for shear coalescence failure to occur, and the cracks formed in the rock bridge region tend to be tensile cracks. Therefore, specimens with a joint angle of 75–90° have the highest percentage of tensile microcracks. The tensile microcracks of the specimen with a joint angle between 15° and 75° show a tendency to increase and then decrease throughout the loading process. Specifically, for joint angles of 15°, 30°, and 75°, the tensile microcrack rises again to a higher level after the drop. While it fluctuates at a lower level or even continues to fall after the drop when the joint angle is 45–60°, consistent with the macro failure characteristics.

## 5. Conclusions

(1) Mechanical parameters of specimens tend to decrease and then increase with the increase of joint angle. The lowest mechanical parameters are found at the joint angle of 30°, where the peak strength is 33.48 MPa, the corresponding strain is 10.35 × 10^−3^, and the elastic modulus is 5.45 GPa. At the joint angle of 90°, the peak strength reaches 44.93 MPa, the corresponding strain is 11.70 × 10^−3^, and the elastic modulus is 5.95 GPa.
(2) According to the failure characteristics of the specimens, the failure modes can be classified into three categories. For a 0–30° joint angle, specimen failure is dominated by tensile failure. When the joint angle rises to 45–60°, the failure plane has obvious shear failure characteristics. For specimens with a joint angle of 75–90°, the failure plane is almost parallel to the loading direction. The percentage of tensile microcracks of the specimen is above 80% when the joint angle shifts from 0° to 30°, and it then decreases with the increase of joint angle, which is mainly attributed to the increase of cracks caused by shear slip.
(3) As joint angle increases, the driving displacement field of wing crack initiation in the joint tips shifts from direct tensile displacement field to indirect tensile displacement field. When the joint angle is located at 0–30°, the wing cracks initiated from the upper joint are driven by a direct tensile displacement field. For specimens with a joint angle of 45–60°, the wing cracks may be actuated by both the direct and the indirect tensile displacement fields. Among the coplanar two joints, the initiation of a wing crack at the tip of the joint in the hard layer is usually caused by an indirect tensile displacement field. For the other two joints, downward-expanding wing cracks tend to be controlled by direct tensile displacement fields, while the driving displacement field of upward-expanding wing cracks is related to the joint angle. For specimens with a joint angle of 75–90°, the wing crack initiation is driven by an indirect tensile displacement field.

Due to the large number of joints in natural rock mass, it is difficult to carry out the test of rock mass with three joints to reflect the failure characteristics of rock mass absolutely. In the future, the study of soft-hard composite rock with multiple joints should be considered. The rock is always under triaxial loading conditions, so it is also necessary to carry out experimental or numerical simulation research on soft-hard composite rock with joints under triaxial compression. In addition, adopting statistical theory to study the mechanical parameter trend is also valuable.

## Data Availability

The data used to support the findings of this study are available from the corresponding author upon request.

## Nomenclature

DIC	Digital image correlation
AE	Acoustic emission
ISRM	International Society for Rock Mechanics
CCD	Charge Coupled Device
